# Today’s referral is tomorrow’s repeat patient: referrals to and between medical outpatient clinics in a hospital

**DOI:** 10.1186/s12913-022-07633-y

**Published:** 2022-02-24

**Authors:** Mariam Safi, Robyn Clay-Williams, Bettina Ravnborg Thude, Julija Vaisman, Frans Brandt

**Affiliations:** 1 Internal Medicine Research Unit, University Hospital of Southern Denmark, Department for Regional Health Research, Aabenraa, Denmark; 2 University of Southern Denmark, Odense, Denmark; 3 Australian Institute of Healthcare Innovation, Macquarie University, Sydney, Australia

**Keywords:** Inter-departmental referrals, Outpatient clinics, Secondary care, Referrals

## Abstract

**Background:**

Unnecessary referrals in Danish hospitals may be contributing to inefficient use of health services already stretched and under pressure and may lead to delayed treatment for patients. Despite a growing awareness in the literature and in practice of issues related to referrals, there has been relatively little research on referrals between specialists in hospital outpatient clinics and how it can be improved. This study aimed to describe the referral patterns to and within the Medical Department at the University Hospital of Southern Denmark. The Medical Department consists of the following medical specialist outpatient clinics; nephrology, pulmonology, endocrinology, cardiovascular, wound outpatient clinic, and a day hospital.

**Methods:**

Two specialist physicians assessed all referrals to the medical specialist outpatient clinics over one month (from 01 September 2019 to 30 September 2019) using data drawn from the Danish electronic patient record system (Cosmic). Data on referral pattern, and patient age and sex, were statistically analysed to identify and characterise patterns of referral.

**Results:**

Four hundred seventy-one (100%) referrals were included in the study. 49.5% (233) of the referrals were from the hospital and 50.5% (238) from general practitioners (GPs). Of the 233 referrals from the hospitals, 31% (72) were from the Medical Department.

**Conclusion:**

The high rate of referrals (31%) from own Medical Department or outpatient clinics may reflect an inefficient internal referral process within the department. Improved collaboration between specialists could have the potential to improve health outcomes, timely access to care and more appropriate healthcare resource utilisation.

## Introduction

**Fig. 1:**
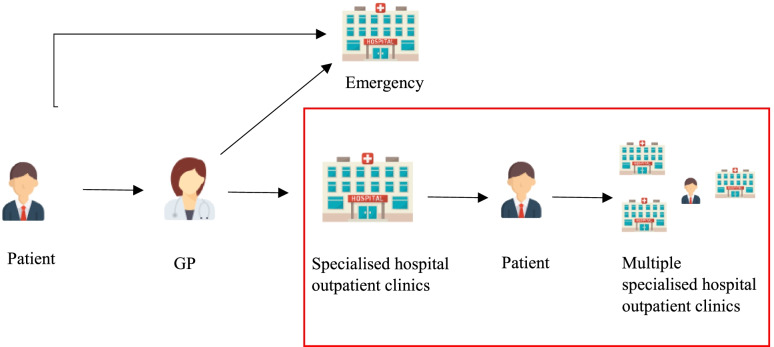
Overview of the referral process in Denmark

Denmark has a universal healthcare system. General practitioners (GPs) act as gatekeepers between the primary level and the specialised healthcare system for non-acute patients (see Fig. 1). When necessary, the GP will refer the patient to specialist hospital services. The specialist can also refer patients to other specialists for diagnoses and treatment. All referrals and communication are made electronically in the Danish healthcare system [1].

Previous studies show that both patients and healthcare professionals find care to be “single-disease” oriented, resulting in gaps in continuity of care for referrals among multiple specialised hospital outpatient clinics (see Fig. 1) [2–5]. With the expected increase in the volume of activity in specialty care [6, 7] due to demographic challenges [8], but without a commensurate increase in resources to meet demand, optimisation of referral processes may ensure efficient patient flow and use of resources. Both referral rate and process have significant implications for patients, the healthcare system, and healthcare costs [9]. An inefficient referral process can be associated with over testing and repetitive testing [10–12], delayed treatment [13, 14], patient confusion regarding navigation of patient pathways [10, 14], ineffective use of physician and hospital services [11, 15], and impaired patient outcomes [10, 11].

A Danish register study showed that chronic patients managed simultaneously in multiple specialist outpatient clinics accounted for 8.3% of all hospital outpatient visits in 2004 and 15.6% in 2014, displaying an 89% increase over 10 years [6]. Furthermore, there was a proportional association between the number of clinics patients are managed in and their rate of visits [7]. These results suggest that the volume of referrals between outpatient clinics will increase over time. For many patients, coordinating the multiple caregivers and adhering to complex treatments can be time-consuming and overwhelming [14].

**Fig. 2:**
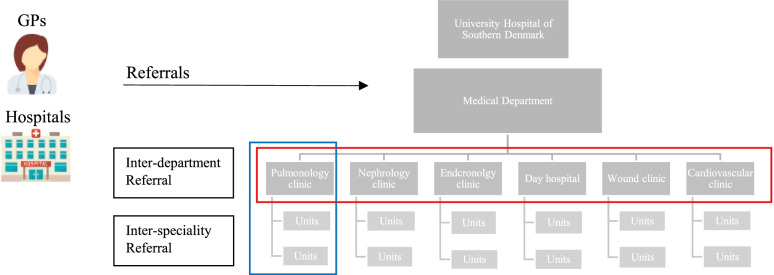
Organisational diagram of the Medical Department and the referral channels

To improve patient access and experience in Southern Denmark, the initial focus was primarily on the referral process from GPs to hospital outpatient clinics [15–17]. More recently, concerns about cost and quality of care, in addition to patient satisfaction, have increased the need to assess the referrals to and within the specialised hospital outpatient clinics (see Figs. 1 and 2). Over the past few years, the University Hospital of Southern Denmark has invested considerable resources in improving the care and management of patients with multi-morbidity, including planning a new workflow within the Department of Medicine. A quality improvement initiative was undertaken to assess the need for improvement initiatives, such as joint patient triage and joint medical outpatient clinic, at the hospital to provide opportunities for inter-professional collaboration. We hypothesised that the need for a joint or collaborative patient triage was necessary because; 1) GPs refer patients based on their symptomatic picture rather than a confirmed diagnosis, and 2) a higher proportion of the referred patients from GPs are also scheduled to attend one of the other outpatient speciality clinics at the hospital, than patients referred by specialists. While there is plentiful information about the referral process from primary care to secondary care [15–20], the information on the inter-department and inter-speciality referral processes are inadequate to support appropriate referrals between specialists (see Fig. 1).

The study aimed to describe the referral pattern to, and between, the medical specialist outpatient clinics in the University Hospital of Southern Denmark (see Fig. 2). Additionally, to explore differences in the nature of internal and external referrals. Specifically, the objectives are:

i. To determine the referral rates to the medical specialist outpatient clinics from GPs and other hospital outpatient clinics (external referral).
ii. To determine if the referred patients from GPs have also attended one of the other outpatient clinics at the hospital.
iii. To determine the inter-department and inter-speciality referral (internal referral) rates between the medical specialist outpatient clinics.

## Method

### Setting

This study was conducted at the Medical Department at the University Hospital of Southern Denmark. The Medical Department consists of five medical outpatient clinics (nephrology, pulmonology, endocrinology, cardiovascular, wound outpatient clinic) and a day hospital (a department in the hospital that provides same-day treatment) (see Fig. 2). These settings were selected to participate in the study because they are undergoing major structural changes to improve workflow and inter-departmental care planning for patients managed in multiple specialist outpatient clinics through joint initiatives.

Inclusion criteria were any referral to one of the medical specialist outpatient clinics at the University Hospital of Southern Denmark which the patient is not currently attending. We were interested in obtaining a comprehensive overview of the referrals. Furthermore, we had initially hypothesised that patients referred by GPs were also scheduled to attend another outpatient speciality clinic at the hospital. We included adult patients over 18 years of age.

Definitions relevant to the study are as follows:

- Internal referral. Internal referrals are inter-departmental and inter-speciality referrals.
  An inter-departmental referral is any referral between the medical specialist outpatients clinics included in the study, such as the endocrinology outpatient clinic referring patients to the nephrology outpatient clinic (see Fig. 2).
  An inter-speciality referral is any referral within its own speciality clinic, such as the pulmonology outpatient clinic referring patients to themselves (see Fig. 2).
- External referral. An external referral is any referral from GPs and other hospital outpatient clinics to one of the medical specialist outpatient clinics at the University of Southern Denmark which the patient is not currently attending.

### Data collection

The Danish hospitals collect information daily (such as visits, admissions, etc.) from electronic patient medical records. The data are linked with the 10- digit personal identification number (CPR number). A list of CPR numbers of patients aged 18+ years, who were referred to the medical specialist outpatient clinics during 01 September 2019–30 September 2019, were retrieved. The data collection period was randomly selected. Using the CPR number, two specialised physicians extracted referral information on each patient from the Cosmic electronic medical record system [21]. The dataset for extraction was agreed upon during prior discussions between the two physicians, and guided by the inclusion criteria. In cases of uncertainty, the physicians discussed the data points until consensus was achieved. As illustrated in Table 1, the following information was collected for each referral:

**Table 1:** Description of referral data

1. Referral date 2. Referral place (e.g. GP, hospital, medical outpatient clinics) 3. Simultaneous referrals 4. Previous attendance 5. Referrals with ongoing treatment 6. Age and sex	

In line with the Danish data protection law and regulation, all data in Cosmic is stored for 10 years.

### Statistical analysis

Pearson’s X^2^ test was used to investigate significant differences in sex, age and outpatient clinics between internal and external referral sources. The results are summarised and presented in tables and pie chart figures. These facilitate an in-depth interpretation of the data. All analyses were performed using Stata version 17 [22]. For all analyses, *P*-values ≤0.05 were considered statistically significant.

## Results

A total of 471 referrals were included in the study. The age and sex distribution of the patients referred are summarised in Table 2. There were no significant differences between age and sex distribution. Women (49.7%) and men (50.3%) were almost equally represented in the internal and external referral sources. In the internal referrals, the median age was 63 years (interquartile range (IQR) 52.5–77) and in the external referrals, the median age was 65 (IQR 51–74). In both groups, more than 50% of the patients were older than 60 years of age.

**Table 2:** Number of patients referred distributed by sex and, age, and stratified by internal and external referral sources

	Total	Internal	External	*P*-value^a^
	*N* = 471	*N* = 72	*N* = 399	
Sex				0.22
Female	234(49.7%)	31(43.1%)	203(50.9%)	
Male	237(50.3%)	41(56.9%)	196(49.1%)	
Age (years)				0.49
18–29	27(5.7%)	1(1.4%)	26(6.5%)	
30–39	27(5.7%)	3(4.2%)	24(6.0%)	
40–49	54(11.5%)	7(9.7%)	47(11.8%)	
50–59	88(18.7%)	16(22.2%)	72(18.0%)	
60–69	92(19.5%)	14(19.4%)	78(19.5%)	
70–79	112(23.8%)	16(22.2%)	96(24.1%)	
80–89	63(13.4%)	14(19.4%)	49(12.3%)	
90 +	8(1.7%)	1(1.4%)	7(1.8%)	

^a^Pearson’s X^2^ test for group differences between internal and external

The distribution of the referred patients to the medical outpatient clinics is shown in Table 3. There were significant differences between the groups (*p* < 0.001). In the internal group, the majority of the patients were referred to the day hospital (36.1%) followed by pulmonology (27.8%) and cardiovascular (18.1%) outpatient clinics. Pulmonology (35.4%) and cardiovascular (26.1%) outpatient clinics together received more than 50% of the referrals from external referral sources.

**Table 3:** Number of patients referred distributed by outpatient clinics and stratified by internal and external referral sources

	Total	Internal	External	*P*-value^a^
Outpatient clinics	*N* = 471	*N* = 72	*N* = 399	< 0.001
Pulmonology	160 (34.0%)	20(27.8%)	140(35.1%)	
Cardiovascular	117(24.8%)	13(18.1%)	104(26.1%)	
Endocrinology	77(16.3%)	8(11.1%)	69(17.3)	
Day hospital	63(13.4%)	26(36.1%)	37(9.3%)	
Wound	29(6.2%)	2(2.8%)	27(6.8%)	
Nephrology	25(5.3%)	3(4.2%)	22(5.5%)	

^a^Pearson’s X^2^ test for group differences between internal and external

**Fig. 3:**
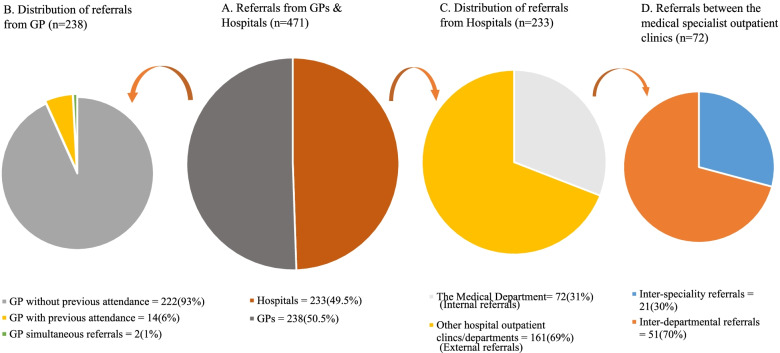
Pie chart showing the sources of referrals

As illustrated in Fig. 3A, referrals where equally distributed from hospitals 49.5% (233) and GPs 50.5% (238). Of the 238 referred patients from GPs, 6% [14] were also attending another speciality, and 1% [2] were simultaneous referrals, such as the GP referring the patient to two outpatient clinics at the same time (See Fig. 3B). The remaining 93% (222) of the referrals were not scheduled to attend another speciality.

Of the 233 referrals from hospitals, 31% (72) were referred from the Medical Department (internal referrals) and the remaining are external referrals (See Fig. 3C). Of the 72 referrals from the Medical Department, 70% (51) were inter-department referrals, such as nephrology outpatient clinic referring patients to endocrinology outpatient clinic. The remaining 30% [21] of the 72 referrals from the Medical Department were inter-speciality referrals e.g. pulmonology outpatient clinic referring patients to their own pulmonology outpatient clinic, meaning clinics referring patients to themselves (See Fig. 3D).

The colours in Fig. 3 are used to separate the categories. The arrows illustrate the distribution of referrals in different categories.

## Discussion

We sought to describe the referral pattern to, and between, the medical specialist outpatient clinics in the University Hospital of Southern Denmark. Our study showed that referrals were evenly distributed between hospitals 49.5% (233) and GPs 50.5% (238), with a very small percentage either attending another speciality or simultaneously referred. Of the referrals from hospitals, one-third (72) were from the Medical Department (internal referrals). This may reflect an inefficient referral process within the department. Of these, inter-departmental referrals accounted for 70% (51) and inter-speciality referrals accounted for 30% [21], meaning that some of the outpatient clinics in the Medical Department referred patients to themselves (self-referral) e.g. pulmonology outpatient clinic referring the patient to their own clinic. Interestingly, day hospital and pulmonology outpatient clinics received the majority of the referrals from internal sources, whereas cardiovascular and pulmonology outpatient clinics received the majority of the referrals from external referral sources.

There is considerable information on referral from GPs in the primary care sector to secondary care internationally [13, 15–19, 23]. Unlike in the current study, these studies [18, 19, 23] showed that the majority of referrals came from GPs. Based on the aforementioned findings that GPs often refer patients according to their symptomatic picture rather than with a confirmed diagnosis [17, 24, 25], we expected that our findings would also comprise of a considerably higher proportion of patients referred from GPs. Additionally, the finding that the Medical Department also had considerably higher proportions of inter-departmental and inter-specialty referrals was unexpected. We propose several reasons for the increased proportion of inter-departmental referrals in our study, including increasing numbers of patients with multi-morbidities that require complex care from several specialists [7, 8] and the internal referral procedure at the Medical Department between the outpatient clinics. However, the unusually high rate of inter-departmental referrals requires further investigation. Furthermore, many previous studies of referral have tended to use self-reported data or survey formats [19, 20, 26–28] to describe referral patterns and examine factors influencing healthcare professionals’ referring habits [4, 29]. There is a possibility that these previous findings may have been impacted by recall bias. Use of register data provides more accurate findings.

Despite the growing awareness of issues related to referrals, there has been relatively little research internationally on referrals between specialists in hospital outpatient clinics or departments. The literature on referral is diverse and differences in contexts, study methods, and measurement of referral patterns make it difficult to compare results between studies. Similar to our study aim, a qualitative study by Burkey et al. [30] on inter-departmental referrals in a hospital indicated that inter-departmental referrals are likely to increase among specialists. Multi-morbidity may explain the same. Furthermore, inefficient referral processes have been considered to play a role in unnecessary referrals resulting in patients undergoing unnecessary diagnostic procedures [10, 11].

The Medical Department at The University Hospital of Southern Denmark in our study is a typical representation of hospitals on an international level that face similar challenges of increasing demands and limited resources. It receives a high proportion of inter-departmental and inter-speciality referrals. It is difficult to explain what causes the inter-speciality referrals. However, a subjective reflection based on clinical experience from the chief consultant and manager of the Medical Department, suggests that the inter-speciality referral or self-referring is often associated with outpatient clinics that are organised into small specialised teams or clinics that have local functions at the hospital’s other facilities. As a result of the organisational system and varying models of care within the same department, the physicians occasionally send formal referrals to their own outpatient clinic when patients require other services from a different team, instead of booking the patient directly for the next appointment. This is further illustrated in that the day hospital received the majority of the referrals from internal sources. Additionally, less experienced or junior physicians may feel more compelled to refer patients through the formal channels because they may be unfamiliar with the referral procedure. In future research, it would be very interesting to investigate in-depth the differences between e patients being referred internally compared to those being referred from external sources.

Unnecessary referrals may increase the cost of providing care, consuming healthcare resources that could have been used to provide other services [15]. For example, the practice exacerbates the administrative workload for the healthcare professionals and administrative employees such as secretaries that are responsible for screening and sending referrals as well as booking tests for the preliminary appointments. Costs for patients may include delayed treatment and waste of time [31] related to unnecessary patient appointments and repetitive tests [10, 11]. Visiting hospitals several times a year for further assessment could subject patients to stress and affect their mental well-being [14, 31].

A new way of managing referrals is required in the hospital system. Several strategies can be implemented to improve the referral process within the Medical Department, and thereby, reduce high inter-department and inter-speciality referral rates. Measures such as IT-solutions [32], joint triage of referrals [12, 33–35], and co-location [36, 37] of the medical outpatient clinics to create a joint medical outpatient clinic could provide opportunities for inter-professional collaboration due to close physical proximity. Close proximity may stimulate more informal consultation between medical outpatient clinics replacing formal referrals. This would allow specialists to work together availing of information-sharing and diagnosing and treating mutual patients. IT-software can be used to gain an improved overview of patient flow and management [38, 39]. The University Hospital of Southern Denmark already uses IT-software for management and workflow on in-patient wards. Similarly, healthcare professionals in outpatient clinics could use IT-software to communicate with each other and book patients directly for appointments and tests thus, improving the inter-departmental and inter-speciality referral process [38, 39]. Added benefits to using IT-software are easier to locate colleagues and fewer interruptions. Co-location, in combination with IT-software and joint triage of patient referrals, could provide patients with convenient access to necessary diagnostics and allow for same-day treatment from several providers simultaneously. This could reduce ineffective use of physician and hospital services allowing patient care in hospitals to be organised around patient needs rather than the needs of medical specialties involved. Additionally, it may ensure improved workflow and safe ongoing care.

### Implications for practice and future research

We anticipate that our results will be useful for clinicians, managers, decision-makers, and researchers in understanding and improving referral processes. Our study sheds light on potential inefficiency in referral processes within a hospital, which can be explained by the organisational structure and the administrative system. Our findings could stimulate similar practices and hospital systems nationally and internationally to conduct an assessment of their current referral and routine practice to minimise unnecessary referrals and improve inter-departmental collaboration between specialists. The findings may also help guide strategies for quality improvement and serve to initiate discussions on how care of patients between multiple outpatient clinics within a single hospital system is best organised.

### Strength and limitations

While the size of the study was comprehensive, the study was limited to a short period of time and a single health service in a region of Denmark, which may limit the generalisability of our results.

Due to the availability of the data, it was not possible to examine the differences between the sub-groups within the external referral category, which include referrals from different organisations (GPs and hospitals). It would be interesting to investigate how referrals from primary care differ from hospital referrals to the medical department as hospitals are highly specialised in comparison to primary care. GPs generally refer patients to specialised care for diagnostic reasons, whereas referrals from highly specialised hospitals to other specialised hospitals may be part of an on-going treatment plan or a transfer of a patient. Thus it is conceivable that referrals from hospitals are more precise compared to referrals from GPs. It may be possible that the referrals from GPs are completed, to a greater extent, after the first consultation. To improve the referral process, future studies should investigate the characteristics of each of the referral processes.

Additionally, the number of internal referrals may be due to organisational factors, local practices and different models of care and could be resolved by multi- and interdisciplinary care.

A strength of our study is that we used registry data: the Danish hospitals routinely collect data linked to the 10- digit personal identification number (CPR number) and hence provide highly valid and reliable data for research.

## Conclusions

The study revealed that referral practices at the Medical Department at the University Hospital of Southern Denmark are potentially inefficient. Reasons for this may be organisational structure and varying models of care within the same department and requires further investigation. One third of the referrals from the hospitals were from our own Medical Department, meaning high rate of referrals between the medical outpatient clinics and high rate of self-referrals within the specialties. These practices could be improved by simple initiatives such as co-location of outpatient services, joint triage of patient referrals, and the IT-software managements. This would result in eliminating over testing and repetitive tests, unnecessary referrals and patient appointments, which would benefit the patients and the healthcare system. In the future, qualitative studies may be conducted to better understand the inter-department and inter-speciality referral process within the hospital from the healthcare professionals’ perspectives. This may illustrate how the work is actually done in practice, versus how it is imagined. Future research will aim to enhance our understanding of the system, how it is organised, why it generates unnecessary referrals, and exacerbates the administrative workload.

## Data Availability

The study follows the Danish GDPR regulations. The datasets generated during and/or analysed during the current study are available from the corresponding author on request.
